# Behavioral and Neurochemical Effects of Extra Virgin Olive Oil Total Phenolic Content and *Sideritis* Extract in Female Mice

**DOI:** 10.3390/molecules25215000

**Published:** 2020-10-28

**Authors:** Nikolaos Kokras, Eleni Poulogiannopoulou, Marinos G. Sotiropoulos, Rafaella Paravatou, Eleni Goudani, Maria Dimitriadou, Electra Papakonstantinou, George Doxastakis, Despina N. Perrea, George Hloupis, Apostolis Angelis, Aikaterini Argyropoulou, Anthony Tsarbopoulos, Alexios-Leandros Skaltsounis, Christina Dalla

**Affiliations:** 1 Department of Pharmacology, Medical School, National and Kapodistrian University of Athens, Mikras Asias 75, Goudi, 11527 Athens, Greece; nkokras@med.uoa.gr (N.K.); eleni.poulo@gmail.com (E.P.); marinos@mail.com (M.G.S.); rafaellaparavatou@yahoo.gr (R.P.); elgkountani@gmail.com (E.G.); mg.dimitriadou@gmail.com (M.D.); electra_papacon@hotmail.com (E.P.); atsarbop@med.uoa.gr (A.T.); 2 First Department of Psychiatry, Eginition Hospital, Medical School, National and Kapodistrian University of Athens, Vas. Sofias Avenue 72–74, 11528 Athens, Greece; 3 Electronic Devices and Materials Laboratory, Department of Electrical and Electronic Engineering, School of Engineering, University of West Attica, Agiou Spiridonos 28, Egaleo, 12243 Athens, Greece; gdoxastakis.ee@gmail.com (G.D.); hloupis@uniwa.gr (G.H.); 4 Laboratory of Experimental Surgery and Surgical Research N.S. Christeas, Medical School, National and Kapodistrian University of Athens, Mikras Asias 75, Goudi, 11521 Athens, Greece; dperrea@med.uoa.gr; 5 Department of Pharmacognosy and Natural Products Chemistry, Faculty of Pharmacy, National and Kapodistrian University of Athens, Panepistimiopolis Zografou, 15771 Athens, Greece; aangjel@pharm.uoa.gr (A.A.); katarg@pharm.uoa.gr (A.A.); skaltsounis@pharm.uoa.gr (A.-L.S.); 6 Bioanalytical Department, GAIA Research Center, The Goulandris Natural History Museum, Othonos 100, Kifissia, 14562 Athens, Greece

**Keywords:** olive oil, *Sideritis*, monoamines, aminoacids, cognition, behavior

## Abstract

The aim of this study was to determine the cognitive and behavioral effects of extra virgin olive oil total phenolic content (TPC) and *Sideritis* (SID) extracts in female mice, and identify the associated neurochemical changes in the hippocampus and the prefrontal cortex. All animals received intraperitoneal low or high doses of TPC, SID or vehicle treatment for 7 days and were subjected to the Open Field (OF), Novel Object Recognition (NOR) and Tail Suspension Test (TST). The prefrontal cortex and hippocampus were dissected for analysis of neurotransmitters and aminoacids with high performance liquid chromatography with electrochemical detection (HPLC-ED). Both TPC doses enhanced vertical activity and center entries in the OF, which could indicate an anxiolytic-like effect. In addition, TPC enhanced non-spatial working memory and, in high doses, exerted antidepressant effects. On the other hand, high SID doses remarkably decreased the animals’ overall activity. Locomotor and exploratory activities were closely associated with cortical increases in serotonin turnover induced by both treatments. Cognitive performance was linked to glutamate level changes. Furthermore, TPC reduced cortical taurine levels, while SID reduced cortical aspartate levels. TPC seems to have promising cognitive, anxiolytic and antidepressant effects, whereas SID has sedative effects in high doses. Both extracts act in the brain, but their specific actions and properties merit further exploration.

## 1. Introduction

Epidemiological and clinical studies have shown that one of the many health benefits of the Mediterranean diet is its protective effect on the brain [1]. However, the neuropsychopharmacology of its individual components, such as olive oil and herbal tea, remains largely unexplored, as is the case with many natural products and herbal remedies with possible psychotropic and nootropic actions [2].

Virgin (VOO) and extra virgin olive oil (EVOO), which is produced from olive fruits (*Olea europaea*), is a key component of the Mediterranean diet. Besides its preventing effects concerning cardiovascular events, cancer, diabetes and inflammation, it has also been credited as a neuroprotective agent and cognitive enhancer [3]. Olive oil’s phenolic compounds have been implicated as key factors for its beneficial properties, and are commonly extracted, quantified with high performance liquid chromatography (HPLC), and used as an index of the oil’s quality and antioxidant activity [3,4]. The total phenolic compound refers to the EVOO’s biophenol fraction, and is a complex mixture containing more than 30 molecules, including secoiridoids (e.g., oleocanthal, oleacein, oleuropein and ligstroside aglicons, elenolic acid derivatives), simple phenols (e.g., hydroxytyrosol and tyrosol), triterpens (maslinic acid, oleanolic acid), flavonoids (luteolin, apigenin) and phenolic acids [3,4].

*Sideritis* sp. (ironwort, mountain tea, or shepherds’ tea) is a caffeine-free aromatic plant of the Lamiaceae family, and is commonly consumed in Mediterranean and Balkan countries as a herbal tea or condiment with promising healing properties, as it has been found to have anti-inflammatory, antimicrobial, antiulcer and analgesic properties [5,6]. Chemical constituents, such as terpenoids, flavonoids, sterols, iridoids, lignans and coumarins, have been identified in *Sideritis* sp. In the brain, the extract functions as an antioxidant and triple monoamine reuptake inhibitor (for dopamine, norepinephrine and serotonin) [6], it inhibits acetylcholinesterase [7], and, as was recently shown in rodent experiments, it exerts an anxiolytic effect [8]. Additionally, a randomized controlled trial revealed *Sideritis’* anxiolytic and cognitive-enhancing properties in humans, which could result from cerebral blood flow modulation [5].

Both EVOO and *Sideritis* species contain phenolic compounds of various chemical structures, whose antioxidant and other effects have been suggested to prevent cognitive decline [1,3]. Although phenols are commonly referred to as neutraceuticals, the literature on their pharmacology is often contradictory, due to the use of different animal models and various dosages, and the fact that their pharmacodynamics are largely unexplored [9]. Therefore, there is a need for a more detailed investigation of these natural substances as possible anxiolytics, antidepressants and/or cognitive enhancers, in order to safely incorporate them into therapeutics, either as new drugs or as dietary supplements.

Although dementia and most mental disorders, including anxiety and depression, are more common in women than men [10], neuropsychopharmacological experiments commonly use male rodents, resulting in an underrepresentation of females in preclinical studies, and a subsequent call for sex awareness in preclinical neuropsychiatric research [11]. For these reasons, in the present study female mice were used, in order to determine the cognitive and behavioral effects of total phenolic content (TPC) and sideritis (SID) extract, and to identify respective neurochemical changes in the brain.

## 2. Results

### 2.1. Extracts Analysis

**Figure 1:**
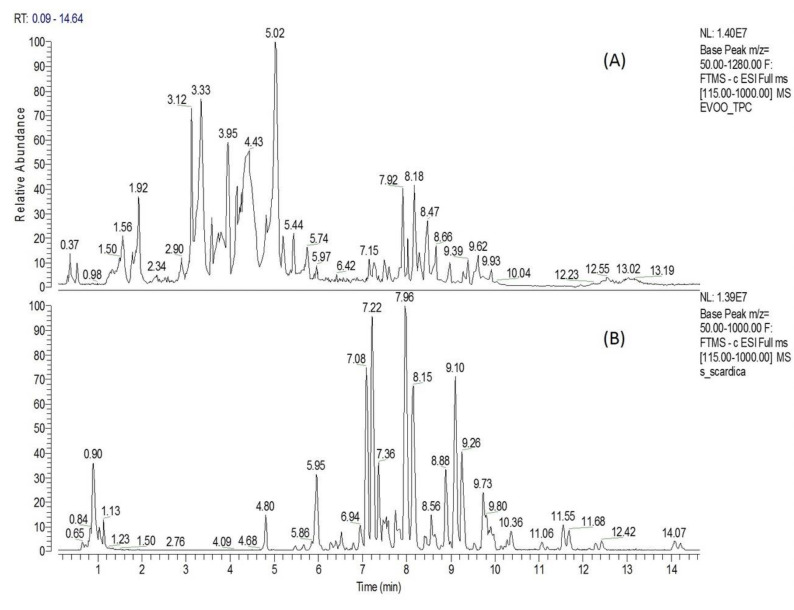
Extracts analysis. Liquid Chromatography-High Resolution Mass Spectrometry (UPLC-ESD-HRMS) chromatogram of extra virgin olive oil biophenolic fraction (TPC) (**A**) and *S. scardica* hydroaloholic extract (SID) (**B**).

Extra virgin olive oil TPC was characterized chemically by Liquid Chromatography-High Resolution Mass Spectrometry (UPLC-ESI-HRMS) analysis. Chromatographic and spectrometric features, such as retention time (tR), suggested formula, RDBeq, MS/MS fragmentation pattern, as well as comparison with the current literature’s data (Olmo-García L. et al., 2018; Angelis A. et al., 2020; Angelis A. et al., 2018), were used to tentatively identify the majority of TPC components. The base picks from the TPC chromatogram revealed a rich content of compounds, especially between 1 and 11 min (Figure 1A). The above analysis led to a tentative identification of 39 metabolites, which belong mainly to six chemical categories, i.e., secoiridoids, simple phenols, triterpens, flavonoids, phenolic acids and fatty acids (Appendix A, Table A1). Secoiridoid derivatives represent the main chemical category of TPC extract, covering 16 of the total of 39 identified metabolites (Table A1: No. 6, 7, 11–12, 15–17, 19–20, 22–24, 27–29 and 31). Among them, elenolic acid, oleacein, oleocanthal and MFLA were the most abundant, presenting with sharp peaks on the TPC chromatogram at *t* = 3.33, 3.79, 4.43 and 5.02 min, respectively (Figure 1A). Moreover, four simple phenolic compounds (3,4-dihydroxyphenylglycol, hydroxytyrosol, tyrosol and hydroxytyrosol acetate), three flavonoids (luteolin, apigenin and diosmetin), three triterpenic acids (maslinic, oleanolic and betulinic acid), seven organic/phenolic acids (Table A1: No. 1–2, 4, 8–10, 18) and six fatty acids (Table A1: No. 30, 32, 34–37) were tentatively identified.

On a second analysis level, the amounts of the high-value TPC constituents (oleocanthal, oleacein, hydroxytyrosol and tyrosol) were determined by HPLC-DAD quantitative analysis. The results of the quantitative analysis (expressed in mg/g TPC) showed that TPC contained 47.6 mg/g oleacein, 122.3 mg/g oleocanthal, 16.4 mg/g hydroxytyrosol and 20.8 mg/g tyrosol.

The UPLC-ESI-HRMS analysis of *S. scardica* hydroaloholic extract revealed a rich phytochemical profile, resulting in the tentative identification of 40 components. The identification process was based on suggested elemental composition (EC), RDBeq values, HRMS/MS spectra and the literature data (Axiotis E. et al., 2020; Petreska J. et al., 2011). The total ion chromatogram of the above extract (Figure 1B) showed a plethora of compounds (especially between 4 and 12 min), which seem to mainly belong into three chemical categories, i.e., hydroxycinamic derivatives, phenylethanoid glucosides and flavonoid glucosides (Appendix A, Table A2). Flavonoid glycosides represent the most abundant chemical category of SID extract, covering 25 of the 40 identified metabolites. Among them, six contain an apigenin skeleton (No. 6, 21, 23 and 34–36; Table A2), six scutellarein and isoscutellarein (No. 13, 22, 24, 31, 32 and 37; Table A2), three a luteolin skeleton (No. 14, 30 and 38; Table A2), nine a hypolaetin skeleton (No. 16, 17, 26–29, 33, 39 and 40; Table A2) and one is chrysoeriol glucoside (No. 25; Table A2). It is important to note that three isoscutellarein glycosides (Figure 1B: t_R_ = 7.36, 7.97 and 9.10) and two hypolaetin glycosides (Figure 1B: t_R_ = 8.15 and 9.26) presented as major peaks in the total ion chromatogram. Moreover, the above analysis led to the identification of nine phenylethanoid glycosides (No. 8–12, 15 and 18–20; Table A2), of which lavandulifolioside and verbascoside are underlined as major SID constituents (Figure 1B: peaks at t_R_ = 7.08 and 7.22, respectively). Furthermore, three hydroxycinamic derivatives were identified (No.4, 5 and 7; Table A2), from which chlorogenic acid seems to be a major SID constituent (Figure 1B: peack at t_R_ = 5.95).

### 2.2. Open Field Test

**Figure 2:**
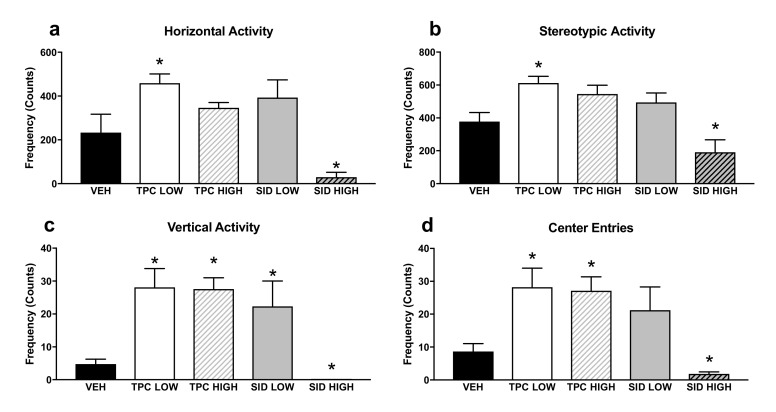
Open Field test horizontal (**a**), stereotypic (**b**) vertical (**c**) activity and center entries (**d**) in the Open Field. TPC Low Dose = 10 mg/kg, High Dose = 30 mg/kg, SID Low Dose = 50 mg/kg, High Dose = 150 mg/kg. N = 7–9/group. An asterisk denotes a significant difference between a treatment group and the corresponding vehicle-treated control group.

Concerning the Open Field (OF) test (Figure 2), there was a significant treatment effect for horizontal counts (F_(4,36)_ = 16.006 *p* < 0.001)), and posthoc pairwise comparisons showed that the low, but not the high, TPC dose increased the ambulatory counts (*p* = 0.001). Inversely, the SID high dose and not the low dose decreased the ambulatory counts (*p* = 0.001).

There was another significant treatment effect for vertical counts (F_(4,36)_ = 17.727 *p* < 0.001)), and post-hoc pairwise comparisons showed that both low and high TPC doses, as well as the low SID dose, increased vertical counts (*p* = 0.001; *p* = 0.001; *p* = 0.03, respectively), but the SID high dose reduced the vertical counts (*p* = 0.017).

A significant treatment effect was also found for stereotypic counts (F_(4,36)_ = 8.137 *p* < 0.001)), and post-hoc pairwise comparisons showed that the low, but not the high, TPC dose increased this parameter (*p* = 0.013). Inversely, the high but not the low SID dose tended to decrease stereotypic counts (*p* = 0.088).

Finally, there was also a significant treatment effect for center entries (F_(4,36)_ = 10.699 *p* < 0.001), and post-hoc pairwise comparisons showed that both the low and high TPC dose increased the center entries (*p* = 0.020; *p* = 0.024, respectively). The SID low dose did not induce any changes, but the SID high dose decreased the center entries (*p* = 0.024).

### 2.3. Object Recognition Test

**Figure 3:**
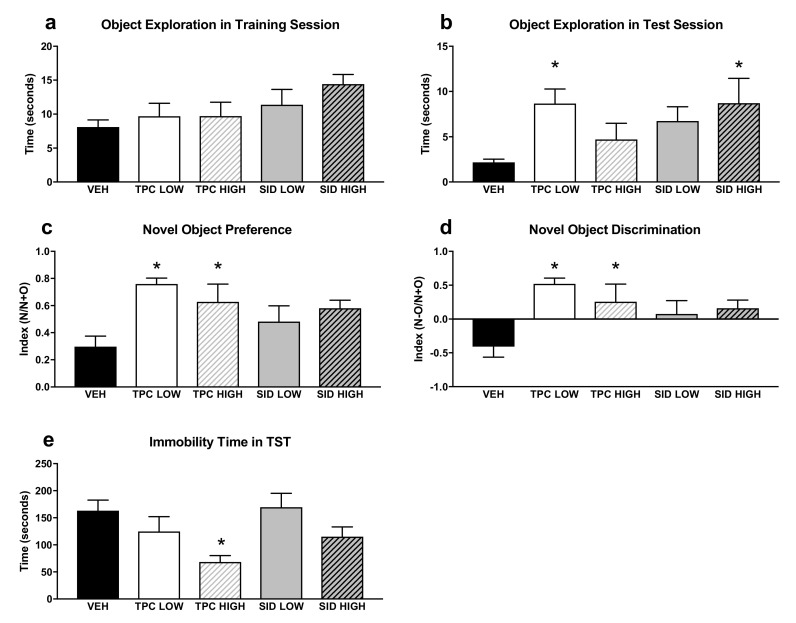
Novel Object Recognition test (NOR) and Tail Suspension Test (TST). (**a**) Total time of object exploration in the first training NOR session. (**b**) Total time of exploration in the second NOR test session. (**c**) Preference index for the new object in the second NOR test session, calculated as N/(N+O), where N = new object and O = old object. (**d**) Discrimination index in the second NOR test session, calculated as (N-O)/(N+O). (**e**) Immobility time in the TST, calculated by the automated algorithm (see Methods). TPC Low Dose = 10 mg/kg, High Dose = 30 mg/kg, SID Low Dose = 50 mg/kg, High Dose = 150 mg/kg. N = 7–9/group. An asterisk denotes a significant difference between a treatment group and the corresponding vehicle-treated control group.

A one-way ANOVA did not show any significant differences induced by TPC and SID treatments for total exploration time of both objects during the training Novel object recognition (NOR) session (Figure 3a–d).

With regard to the test NOR session, a one-way ANOVA showed the significant treatment effect on the total exploration time of both objects (F_(4,36)_ = 2.698 *p* < 0.046)), and post-hoc pairwise comparisons showed that the low dose, but not the high dose, of TPC increased exploration time (*p* = 0.024). Moreover, the SID high dose, but not the low dose, increased the exploration time (*p* = 0.05).

A one-way ANOVA showed a significant treatment effect for the discrimination index (F_(4,36)_ = 4.361 *p* = 0.006), and post-hoc pairwise comparisons indicated that the TPC low and high doses increased the discrimination index (*p* = 0.001; *p* = 0.035). The SID low and high doses did not affect the discrimination index, although there was a trend for the SID high dose to increase it (*p* = 0.084). Similar results were seen when examining the NOR preferences index, given that there was a significant treatment main effect (F_(4,36)_ = 3.695 *p* = 0.013), and post-hoc testing showed that both low and high TPC doses increased the preference for the new object (*p* = 0.003; *p* = 0.038), whereas no preference for the new object was observed after both low and high SID doses.

### 2.4. Tail Suspension Test

A one-way ANOVA showed a significant treatment effect for immobility time (F_(4,36)_ = 3.228 *p* = 0.023), and post-hoc pairwise comparisons showed that the high dose of TPC decreased the immobility time (*p* = 0.019). No differences were observed with other treatments (Figure 3e).

### 2.5. Monoamines in the Hippocampus

**Figure 4:**
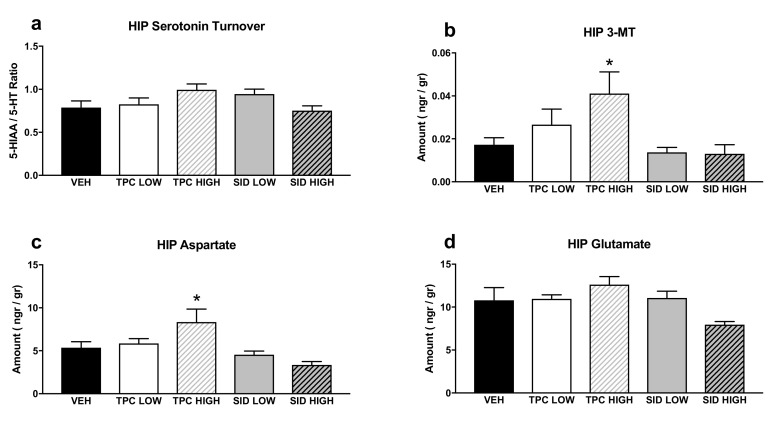
Aminoacids and monoamines in the hippocampus (HIP). (**a**) Serotonin turnover rate, calculated as the 5-HIAA/5-HT ratio; (**b**) 3-MT tissue levels; (**c**) aspartate tissue levels; (**d**) glutamate tissue levels. TPC Low Dose = 10 mg/kg, High Dose = 30 mg/kg, SID Low Dose = 50 mg/kg, High Dose = 150 mg/kg. N = 7–9/group. An asterisk denotes a significant difference between a treatment group and the corresponding vehicle-treated control group.

A one-way ANOVA showed a trend indicating the treatment effect for serotonin turnover (F_(4,36)_ = 2.174 *p* = 0.092), but post-hoc comparisons did not show any significant differences (Figure 4a).

A one-way ANOVA showed a significant treatment effect for 3-MT (F_(4,36)_ = 3.461 *p* < 0.017), and post-hoc pairwise comparisons showed that there was a trend for increased 3-MT after the high dose of TPC (*p* = 0.063). No differences were observed with other treatments (Figure 4b).

No differences were detected in DA turnover rates (HVA/DA and DOPAC/DA ratios), or 5-HT, HVA, 5-HIAA, DA, DOPAC and NA tissue levels (Table A3).

### 2.6. Aminoacids in the Hippocampus

A one-way ANOVA showed a significant treatment effect for aspartate (F_(4,36)_ = 5.154 *p* = 0.002), and post-hoc pairwise comparisons indicated that a high dose of TPC increased aspartate tissue levels (*p* = 0.034), while the other treatments did not induce any changes in aspartate levels (Figure 4c).

A one-way ANOVA showed a significant treatment effect for glutamate (F_(4,36)_ = 2.795 *p* = 0.04), but post-hoc pairwise comparisons did not show any significant differences between each treatment and the vehicle (Figure 4d).

No differences were detected in Gamma aminobutyric acid (GABA), arginine, taurine, glutamine, glycine and serine tissue levels (Table A3).

### 2.7. Monoamines in the Prefrontal Cortex

**Figure 5:**
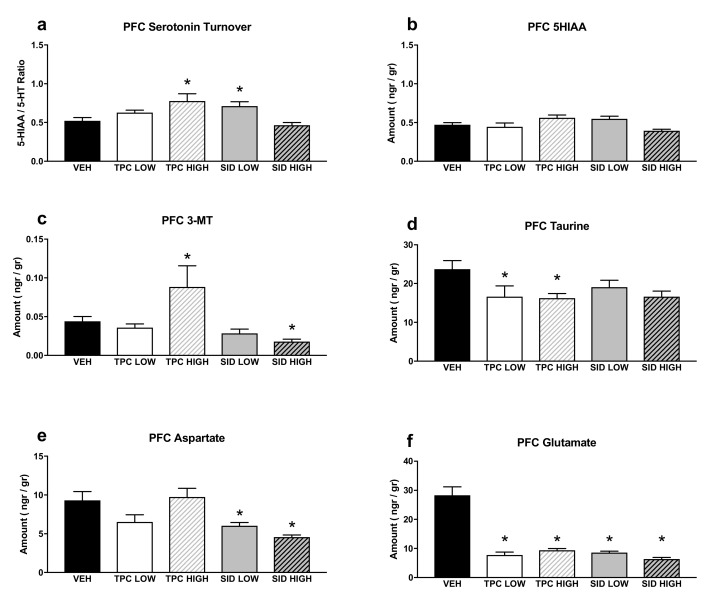
Aminoacids and monoamines in the prefrontal cortex (PFC). (**a**) Serotonin turnover rate, calculated as the 5-HIAA/5-HT ratio; (**b**) 5-HIAA tissue levels; (**c**) 3-MT tissue levels; (**d**) taurine tissue levels; (**e**) aspartate tissue levels; (**f**) glutamate tissue levels. TPC Low Dose = 10 mg/kg, High Dose = 30 mg/kg, SID Low Dose = 50 mg/kg, High Dose = 150 mg/kg. N = 7–9/group. An asterisk denotes a significant difference between a treatment group and the corresponding vehicle-treated control group.

A one-way ANOVA showed a significant treatment effect for serotonin turnover (F_(4,36)_ = 5.770 *p* = 0.001), and post-hoc pairwise comparisons showed that the TPC high dose and the SID low dose increased the serotonin turnover (*p* = 0.008; *p* = 0.031, respectively). No changes were observed following the TPC low dose and the SID high dose (Figure 5a).

A significant treatment effect was noted in a one-way ANOVA for 5-HIAA (F_(4,36)_ = 3.459 *p* = 0.017), but post-hoc pairwise comparisons did not show any differences between treatments and vehicles (Figure 5b).

Additionally, a one-way ANOVA showed a significant treatment effect for 3-MT (F_(4,36)_ = 8.756 *p* < 0.001), and post-hoc pairwise comparisons showed that the high dose of TPC increased and the high dose of SID decreased the tissue content of 3-MT (*p* = 0.036; *p* = 0.012, respectively). No differences were observed with other treatments (Figure 5c).

No differences were detected in the dopamine turnovers (HVA/DA and DOPAC/DA ratios) or the tissue levels of 5-HT, HVA, DA, DOPAC and NA (Table A3).

### 2.8. Aminoacids in the Prefrontal Cortex

There was a significant treatment effect for taurine (one-way ANOVA, F_(4,35)_ = 2.565 *p* = 0.05), and post-hoc pairwise comparisons showed that both low and high doses of TPC reduced taurine tissue levels (*p* = 0.05; *p* = 0.047, respectively). The low dose SID did not cause any changes in taurine levels, whereas there was a trend for lower taurine levels after receiving the high SID dose (*p* = 0.062) (Figure 5d).

A one-way ANOVA showed a significant treatment effect for aspartate (F_(4,35)_ = 6.363 *p* = 0.001), and post-hoc pairwise comparisons showed that both low and high doses of SID reduced aspartate tissue levels (*p* = 0.024; *p* = 0.002, respectively). There was a trend for TPC low dose to reduce aspartate tissue levels (*p* = 0.078), but no changes were seen after receiving the TPC high dose (Figure 5e).

Finally, there was a significant treatment effect for glutamate (one-way ANOVA, F_(4,35)_ = 22.754 *p* < 0.001), and post-hoc pairwise comparisons showed that both doses of TPC and SID reduced glutamate levels (*p* < 0.001 for all treatments vs. vehicle) (Figure 5f).

The same analysis did not reveal significant differences for GABA, arginine, glutamine, glycine and serine, following treatment with either low or high dose of TPC and SID (Table A3).

### 2.9. Serum Chemistry Analysis

One-way ANOVA for AST/SGOT showed a significant treatment main effect (F_(1,35)_ = 5.515 *p* < 0.0010), and post-hoc testing showed that TPC high and low doses reduced serum levels of AST/SGOT (*p* = 0.007; *p* = 0.003, respectively). Likewise, SID low dose reduced AST/SGOT serum levels (*p* = 0.032), but no significant reduction was seen following treatment with the high SID dose (Table 1).

The same analysis showed a trend towards a reduction in ALT/SGPT serum levels (F_(1,35)_ = 2.443 *p* = 0.065), which was mainly because the high dose of TPC tended to decrease ALT/SGPT serum levels (*p* = 0.098) (Table 1).

The analysis indicated a significant treatment main effect on Urea serum levels (F_(1,35)_ = 4.878 *p* < 0.003), and post-hoc testing showed that both the low and the high dose of TPC reduced serum urea levels (*p* = 0.032; *p* = 0.006, respectively), whereas SID treatment had no effect on serum urea levels (Table 1).

No differences were detected in γ-GT and creatinine serum levels, following treatment with either the high or the low dose of TPC or SID.

### 2.10. Multivariate Regression

All behavioral measurements were tested against multivariate regression models, which included the Hippocampus tissue levels of glutamate, aspartate, 3-MT and 5-HT turnover, and the prefrontal cortex tissue levels of glutamate, aspartate, 3-MT, 5-HT turnover, 5-HIAA and taurine, which showed statistically significant results after treatment with TPC or SID. As seen in Table A3, locomotor and exploratory activity (horizontal and vertical activity, respectively) was associated more closely with cortical 5-HT turnover changes induced by treatments, whereas changes in hippocampal and cortical glutamate tissue levels, induced by TPC and SID treatments, were associated more closely with cognitive performance, as measured in the NOR test.

## 3. Discussion

Both olive TPC and SID are mixtures of natural compounds exhibiting antioxidant, neuromodulating and neuroprotective effects [1,3,5,6,8]. This study aimed to investigate their possible activity as anxiolytics, antidepressants or cognitive enhancers. Therefore, their effect on the mood, cognition and behavior of female mice, as well as the accompanying neurochemical changes in the hippocampus and prefrontal cortex, were measured. Two doses of each extract were used, in order to provide adequate dose–response information. However, in the future a more detailed dose–response study could shed further light on the effects of these two extracts.

### 3.1. Behavioral Findings

In this study, the OF novelty test was used as a proxy for the estimation of anxiety [12,13]. TPC administration, in both low and high doses, exerted an anxiolytic effect by increasing exploratory behavior and inducing more center entries. Moreover, only the low TPC doses increased both the ambulatory and the stereotypic counts. TPC affected the overall performance of female mice in the OF, indicating an anxiolytic-like effect. The anxiolytic potency of such phenolic compounds has previously been reported by other groups. Oleuropein, a major phenolic compound in olive leaves and a precursor molecule in the biosynthesis of the major compounds of TPC, displayed anxiolytic-like effects in a model of post-traumatic stress disorder in male rats, both in the OF and the elevated plus maze test [14].

Concerning SID, the low dose did not impact the open field performance in female mice, as expressed by locomotion, stereotypic counts or frequency of center entries. Interestingly, the high dose appeared to remarkably decrease locomotion, stereotypic counts and open field center entries, suggesting a dose-dependent sedative effect in female mice. Vassilopoulou et al., after administering *S. clandestina* in adult male mice (2 and 4% w/v, ad libitum access), observed a dose-dependent anxiolytic-like effect in the OF [8]. However, in our experiment the high SID dose decreased the exploratory behavior and reduced the entries in the central unprotected area, possibly indicating the increased sedative potency of this compound when it is administered in such doses in female mice. Furthermore, the antidepressant potentials of TPC and SID treatments were evaluated with the TST. Interestingly, the high TPC dose decreased immobility time, indicating an antidepressant property of the extract.

Non-spatial working memory was assessed with the NOR test [12,15,16]. While there were no significant treatment effects during the first NOR training session, a significant treatment effect was observed in the second test session: low TPC and high SID doses increased the total exploration time of both objects, whereas high TPC and low SID treatments had no effect on this parameter. Importantly, preference for the new object increased with both low and high TPC doses, indicating improvement in the non-spatial working memory. On the other hand, both low and high SID doses failed to significantly increase these indexes. These results suggest that only the TPC treatment was able to enhance non-spatial working memory. Accordingly, long-term feeding with olive secoiridoids has also been found to exert positive effects on cognition (spatial memory) and brain metabolism in female aged mice [17]. Another study revealed that long-term dietary extra virgin olive oil polyphenols can reverse age-related dysfunctions in motor coordination and contextual memory in male mice [18]. Concerning the SID extract, despite the unpromising results in our experiments in females, its preparations have been found to display memory and learning improvement effects in Alzheimer’s disease mouse models, using aged wild type male mice [19]. Studies in humans point out that long-term *S. scardica* in co-administration with selected B-vitamins results in cognitive improvement in both sexes (alleviates stress-induced impairment of executive functioning, working memory, cognitive flexibility and controlled behavioral inhibition) [20]. Moreover, in subjects with cognitive impairment, the co-administration of *S. scardica* and *Bacopa monnieri* resulted in trending improvements in concentration, arithmetic calculation and memory tasks [21]. In a double-blind placebo-controlled study in healthy males and females, *S. scardica* significantly improved cognitive performance [5].

### 3.2. Neurochemical Findings

**Figure 6:**
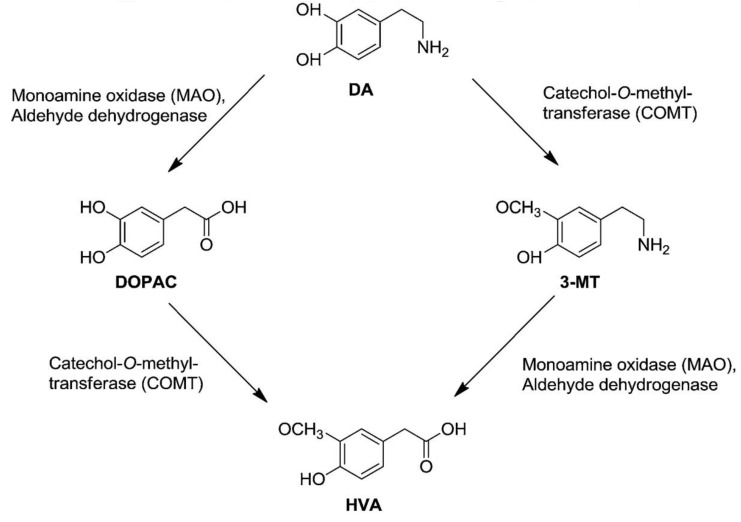
Dopamine Metabolism. Dopamine can be metabolized by aldehyde dehydrogenase (AH), monoamine oxidase (MAO) or catechol-O-methyl transferase (COMT). COMT metabolizes DA to form 3-Methoxytyramine (3-MT), which can be further metabolized by MAO to form homovanillic acid (HVA). Adapted from Gallardo et al., 2014 [22].

In the hippocampi of all treated mice, a treatment effect on 5-HT turnover ratio and 3-MT tissue levels was identified, as high-dose TPC significantly increased hippocampal levels of 3-MT (Figure 4). 3-MT is a dopamine metabolite formed by the enzyme catechol-O-methyl transferase (COMT), and it can be further metabolized by monoamine oxidase (MAO) to form HVA (Figure 6). Ιn this study, increased 3-MT tissue contents could suggest a high-dose TPC-induced increase in COMT activity in the hippocampus. However, this hypothesis cannot be further supported, due to a lack of the expected changes in the DA metabolism; DA turnovers (HVA/DA and DOPAC/DA ratios), as well as DA, DOPAC and HVA tissue levels, remained unaffected.

However, the olive oil phenolic compound hydroxytyrosol and its nitroderivatives have been reported to possibly act as lipophilic COMT inhibitors in the striatum (increased extracellular DOPAC levels, failed to decrease HVA levels below 75%, HIAA levels remained unaffected) [23]. COMT central inhibition is expected to reduce HVA and increase DA levels in the brain (Figure 6), although the significance of extracellular HVA levels still remains unclear. Interestingly, acute or chronic systemic administration of hydroxytyrosol and some of its nitroderivatives increases intracellular HVA, DA and DOPAC levels [22]. However, HVA increase could be attributed to the COMT-induced metabolism of hydroxytyrosol itself to homovanillic alcohol, which is then converted into HVA [23]. Other studies indicate that hydroxytyrosol effectively prevents dopaminergic neuron dysfunction, which is related to DA metabolism [24,25], inhibits both the enzymatic and spontaneous oxidation of endogenous DA [26], and also potently enhances norepinephrine transporter activity in the pheochromocytoma PC12 cells [27]. However, in the present study employing female mice, no differences were detected in the hippocampal whole tissue levels of DA, DOPAC, HVA, 5-HT, 5-HIAA or NA.

In the prefrontal cortex, there was a treatment effect on 5-HIAA tissue levels and 5-HT turnover rates (5HIAA/5-HT ratio). However, the only significant results were found for the high TPC and low SID doses, which increased serotonin turnover rates. Concerning 3-MT, a significant treatment effect was once more identified—the high doses of TPC increased, and the high doses of SID decreased, the tissue content of 3-MT. Dopamine turnovers (HVA/DA and DOPAC/DA ratios) and tissue levels of 5-HT, DA, HVA, DOPAC and NA remained unaffected.

However, it has been reported that *Sideritis*’ flavonoids, xanthomicrol and salvigenin, inhibit hMAO-A selectively and reversibly in a competitive mode. Moreover, it is important to mention that apigenin, one flavonoid compound of *S. scardica*, has been found to enhance, rather than inhibit, monoamine uptake on wild dopaminergic cell lines, with higher specificity for DA than NA and 5-HT [28].

Regarding aminoacids, in the hippocampus, a treatment effect for glutamate and aspartate was observed, and only high-dose TPC increased aspartate levels. Tissue levels of GABA, arginine, taurine, glutamine, glycine and serine remained unaffected. In the prefrontal cortex, both low and high doses of TPC significantly reduced taurine levels. Concerning SID administration, only the high SID doses tended to increase taurine. Taurine has been previously found to affect multiple functions in the central nervous system. This amino acid interferes with calcium currents during depolarization, stabilizing the membrane and exerting anticonvulsant effects. It also participates in learning and memory processes [29,30]. In behavioral experiments, taurine exerts anxiolytic-like effects in rats in the social interaction, hole-board and OF tests, which could be mediated by its interaction with the 5-HT and GABA system, although the exact neurochemical mechanism has yet to be identified [31]. Concerning the OF test, the administration of increasing taurine doses seems to significantly decrease ambulation, latency scores and thigmotaxis, without affecting rearing and defecation [32]. Moreover, a human study using proton magnetic resonance spectroscopy indicated that trait anxiety and state physical fatigue might be negatively associated with nucleus accumbens taurine content [33].

In our experiment, there was also a significant treatment effect for aspartate—both low and high doses of SID extract reduced aspartate tissue levels. A trend for the low TPC dose to reduce aspartate tissue levels was also identified. Moreover, glutamate tissue levels in the prefrontal cortex were significantly reduced by all treatments, but no differences were detected in GABA, arginine, glutamine, glycine or serine tissue levels. Even though SID extract did not affect GABA levels in our experiment, it has been found to enhance GABAergic inhibition by acting as a positive allosteric modulator at GABA-A receptors. [34,35,36]. 

After multivariate regression analysis, two correlations were revealed between the neurochemical and the behavioral effects of the treatments examined. Firstly, locomotor and exploratory behaviors were found to closely associate with cortical 5-HT turnover changes induced by treatments. This correlation, in combination with the enhanced serotonergic ratio in the prefrontal cortex, indicate that the behavioral effects (including the possible antidepressant effect) of high TPC doses may be associated with changes in the serotonergic activity. The molecular mechanisms involved need to be investigated in future studies, but they could be the same as those described for licensed antidepressants, such as serotonin reuptake inhibitors (SSRIs), resulting in enhanced neuroplasticity in the brain and subsequent mood elevation [37,38,39]. It seems that 5-HT modulation is closely related to neuroplastic effects in specific brain regions. Interestingly, serotonergic receptor activation increases neuronal growth factor levels (mostly BDNF), as well as activating the tyrosine kinases involved in cytoskeletal rearrangement or astroglial interactions. Therefore, serotonergic disbalance, a crucial component of depression pathophysiology, might be closely linked to defects in neuroplasticity [39]. Conversely, the neurotrophic actions of antidepressants have been suggested to exert their therapeutic effects by reversing neuronal atrophy and cell loss [38].

Moreover, previous experiments with the antidepressant sertraline have shown that the behavioral response in a test of antidepressant activity correlates with serotonergic changes in the prefrontal cortex, but not the hippocampus [40]. These region-specific results underline the crucial role of the prefrontal cortex in the stress coping mechanism. It is involved in the processing of emotional stimuli, stressor controllability, working memory, flexibility, and the temporal organization of behavior [40].

**Figure 7:**
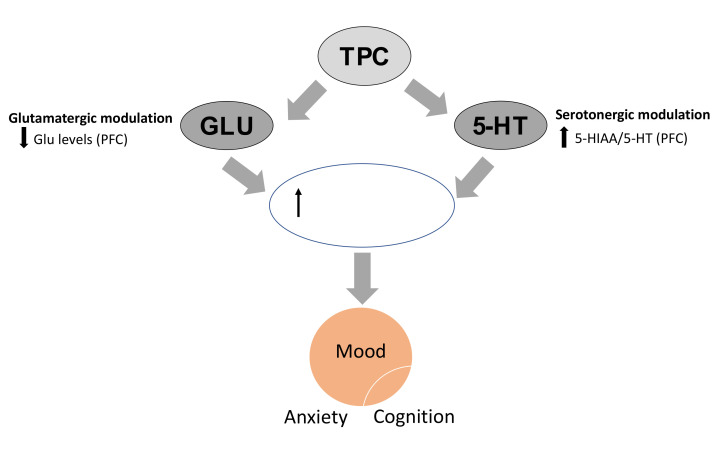
TPC in high doses affects serotonergic and glutamatergic neurotransmission in the PFC that could potentially result in enhanced neuroplasticity, associated with improvements in mood, anxiety and cognition, as it is evident from behavioral results. TPC: total phenolic content, PFC: prefrontal cortex, HIP: hippocampus, 5-HT: serotonin, 5-HIAA: 5-hydroxyindoleatic acid.

Secondly, cognitive performance in the NOR test closely was associated with changes in hippocampal and cortical glutamate levels induced by all TPC and SID treatments. The increase in glutamate levels in the mouse hippocampus (overall effect), in combination with the decrease in the same amino acid in the PFC, could be related to enhanced cognitive performance. Specifically, for the high dose of TPC, this could represent a promising mechanism that in combination with the serotonergic changes could represent a multimodal mechanism of action that could resemble the effect of several licensed antidepressant drugs (Figure 7) [41].

Notably, the glutamate system is the major excitatory neurotransmitter system in the brain and is essential for cognitive processing. Serotonin seems to modulate glutamatergic neurotransmission and might kindle NMDA receptor-dependent plasticity. Several classes of antidepressants, including SSRIs, are known to exert indirect modulating effects on glutamate transmission (e.g., by influencing NMDA or AMPA receptor transcription) [39], thus being considered to treat impaired mood and cognition. Moreover, in the ketamine example, which is a novel rapidly acting antidepressant, it is not clear whether the NMDA receptor blockade alone mediates the antidepressant activity, or if it is an indirect/mixed effect. Interestingly, a recent preclinical report demonstrated that 5-HT depletion abolished ketamine’s antidepressant-like activity, suggesting that 5-HT plays an important role in its action [41].

Furthermore, previous studies have shown that hippocampal and prefrontal DA plays an essential role in cognitive function [42,43,44], and that the hippocampus is a crucial structure for novelty identification [45]. However, in the present study, both the levels and turnover rates of hippocampal DA remained unchanged.

## 4. Methods

### 4.1. Animals

**Figure 8:**
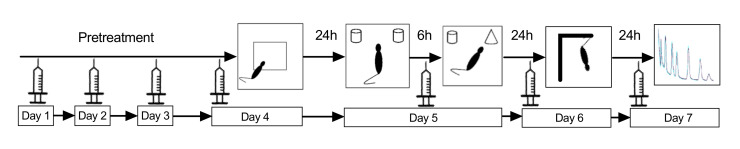
Experimental timeline.

All experiments were conducted in accordance with the guidelines for the care and handling of laboratory animals in EU Directive 2010/63 and were approved by the local ethics committee (Protocol 478/28012014). Female C57BL/6 mice, 6–9 months old, were group-housed under standard laboratory conditions (lights on from 7 a.m. to 7 p.m.; room temperature 22 °C; relative humidity of 55%, ad libitum access to food and water). The timeline of the experiment described below is depicted in Figure 8.

### 4.2. Extract Preparation

TPC was recovered from extra virgin olive oil (EVOO) provided by the Renieris cooperation (Peloponnese, Greece) and produced from olive fruits of “Koroneiki” variety. The liquid–liquid extraction procedure was based on a previously-described multi-dual mode method [46], developed in a centrifugal partition extractor FCPE300^®^ (Rousselet-Robatel Kromaton, Anonay, France) equipped with a 300 mL rotor and connected to a LabAlliance preparative pump (SpectraLab Scientific Inc., Markham, ON, Canada). The EVOO was used as part of the “mobile phase” (n-hexane/EVOO in proportion of 3:2 (v/v)) while the extraction, “stationary phase”, consisted of EtOH/water in proportion of 3:2 (v/v). In total, 20 L of EVOO was treated in several “Extraction–Recovery” cycles resulting in the recovery of 22.6 g of TPC.

*Sideritis scardica* was obtained from local producers. The plant was ground (SCIS, Allenwest-Eac ltd.) into fine homogeneous powder and extracted with ultrasound-assisted extraction (UAE). For that purpose, an Elma S 100H (Elmasonic) was used with water/ethanol (80:20) as the extraction solvent, for 20 min at room temperature. In total, 200 gr of raw material was extracted with 2 L of solvent, and the eluent was evaporated to dryness under reduced pressure using a rotary evaporator (Buchi Rotavapor R-200) at 40 °C resulting in the recovery of 25.9 gr of dry extract.

### 4.3. UPLC-HRMS/MS Qualitative and HPLC-DAD Quantitative Analysis

UPLC-HRMS/MS qualitative analysis of TPC and SID was performed on an ACQUITY system (Waters) connected to an LTQ-OrbitrapR XL hybrid mass spectrometer (Thermo Scientific) equipped with an electrospray ionization (ESI) source and operated in negative mode. Due to the different chemical compositions of TPC (unipolar and middle polarity compounds) and SID (middle polarity and polar compounds), two different UPLC separation gradients were developed in order to efficiently resolve all compounds for the qualitative analysis. The chromatographic separation of TPC was carried out on a Fortis C18 (100 × 2.1 mm, 1.7 μm) column at a temperature of 40 °C, using as a mobile phase a gradient of water (A) and acetonitrile (B) containing 0.1% (v/v) formic acid each. The flow rate was set at 0.4 mL/min and the injection volume at 5 mL. The elution program was set as the following: 98% A decreased to 0% A in 9 min. The same conditions were maintained for 2 min before returning in 1 min to the initial conditions for a 3 min re-equilibration (total run time of 15 min). For the SID separation the flow rate was set at 0.4 mL/min and the solvent system was (A) water 0.1% formic acid and (B) acetonitrile. The elution program was: 2% B for 2 min; 100% B for 18 min; and hold for 2 min. After a return to 2% B for 1 min, column equilibration was performed for 4 min at the end of the run. The injection volumes for both extracts were set to 10 μL and samples were injected at 0.2 mg/mL on a Supelco Ascentis Express C18 (100 × 2.1 mm i.d, 2.7 μm particle size).

The HRMS and HRMS/MS data were acquired in negative mode over a 100–1000 m/z range. The MS profile was recorded in full scan mode (scan time = 1 micro scans and maximum inject time = 500 ms). The ESI conditions were as follows: capillary temperature 320 °C; capillary voltage −40 V; tube lens −120 V; ESI voltage 2.7 kV. Nitrogen was used as the sheath gas (40 Au) and auxiliary gas (8 Au). For the HRMS/MS acquisitions, a data-dependent method including the detection (full scan) and fragmentation of the 3 most intense peaks per scan was used. The mass resolving power was 30,000 for both levels and the normalized collision energy (CID) in the ion trap was set to 35.0% (q = 0.25) for the HRMS/MS experiments. Chromatographic and spectrometric features were used for the identification of extract constituents, such as retention time (Rt), polarity, accurate m/z, proposed elemental composition (EC) and ring double bond equivalent (RDBeq) values, as well as HRMS/MS spectra and derived fragmentation motifs. The raw data were acquired and processed with XCalibur 2.2.4 (Thermo Scientific).

Quantitative analysis of the main bioactive TPC constituents (oleocanthal, oleacein, hydroxytyrosol and tyrosol) was performed on a Thermo Finnigan HPLC system (San Jose, CA, USA) equipped with a SpectraSystem P4000 pump, a SpectraSystem 1000 degasser, a SpectraSystem AS3000 automated injector and an UV SpectraSystem UV6000LP detector monitored at 235, 280 and 365 nm. Data acquisition was controlled by the ChromQuestTM 5.0 software (ThermoScientificTM). The analysis was run in a Discovery HS C18 (15cm × 4.6mm, particle size 5 µm) chromatographic column (Supelco, Bellefonte, PA, USA) at room temperature, using a previously-described HPLC protocol [47]. This protocol includes the use of two different methods, one in non-acidic conditions (method A) for the quantification of oleocanthal and oleacein, and another one in acidic conditions (method B) for the quantitative analysis of hydroxytyrosol and tyrosol. Method A used mixtures of water and acetonitrile as the mobile phase in the following gradient elution: 0–20 min from 80% water to 70%, 20–35 min remaining stable to 70% water, and 35–40 min from 70% water to 80% (flow rate, 1 mL/min). Method B used mixtures of water acidified with 0.2% acetic acid and acetonitrile in the following gradient system: 0–40 min 2–30% acetonitrile, 40–45 min 30% acetonitrile, 45–50 min 30–2% acetonitrile (flow rate, 1 mL/min). The injection volume for both methods was 20 µl (from a solution of 1 mg TPC/mL MeOH), while syringaldehyde (98% HPLC; ExtraSynthese, Genay Cedex, France) was used as the internal standard. Four calibration curves were created (concentrations 50, 100, 200, 300, 400, 500, 600, 700, 800, 900 and 1000 µg/mL for oleocanthal and oleacein, and 2, 3, 20, 50 and 100 µg/mL for hydroxytyrosol and tyrosol) and used to calculate the percentages of the target compounds in TPC.

### 4.4. Treatments

TPC and SID extracts were dissolved in 0.9% NaCl and 35% DMSO. All treatments were volume- and weight-corrected. The injection volume was 10mL/kg and the resulting DMSO exposure (35% v/v DMSO equaling 3.5 mL/kg) was low, and below the recommendation to not exceed 25% of the LD50, which has been found to be 14–20 mL/kg in mice [48]. As a result, no vehicle-related toxicity effects were observed, nor any animal suffering, in accordance with previous observations [49,50,51] and previous studies involving water-insoluble treatments [52,53,54]. The composition of the dissolved extracts, as prepared for animal treatment, was confirmed by HPLC-UV and LC-MS (Figure 1; Table A1 and Table A2). TPC was administered intraperitoneally, once daily, in two doses, 10 mg/kg (TPC LOW dose) and 30 mg/kg (TPC HIGH dose), which were considered safe based on toxicological data (see Appendix A). The SID extract was also administered once daily intraperitoneally in two doses, 50 mg/kg (SID low dose) and 150 mg/kg (SID high dose). All animals received either TPC or SID extract or vehicle treatment for 7 days.

### 4.5. Behavioral Measurements

#### 4.5.1. Open Field Test

On the first day of behavioral testing, 30 min after TPC, SID or vehicle administration, all animals were tested in the open field. The OF test was used to measure locomotor and exploratory behavior (horizontal and vertical counts, respectively) and center entries as an index of anxiety in mice [12,13]. The OF was a plexiglass chamber (W40 × D40 × H40; Med Assoc., Fairfax, VT, USA) with automatic movement detection. Individual mice were introduced into the center of the OF and were allowed to move freely within it for 10 min. Afterwards, they were returned to their home cages. The OF arena was cleaned thoroughly with 70% ethyl alcohol before the next mouse was tested. Behavior during each trial was video recorded and locomotor activity, exploratory behavior and number of entries in the center of the open field were scored using Activity Monitor software (version 5, Med Associates, Fairfax, VT, USA), as described previously [55,56].

#### 4.5.2. Novel Object Recognition Test

On the second day of behavioral testing, the NOR test was used to assess non-spatial working memory, a component of cognition [12,15]. Animals were left to familiarize themselves with the testing room for 1 h, and were then introduced into the NOR apparatus, which consisted of the open field (OF, described above) with the addition of bedding in order to mimic the familiar cage environment, as well as 2 objects placed near the two corners of the open field. Mice were placed at the center of the apparatus during 2 trials of 10 min each. Between the two trials, all mice received either SID, TPC or vehicle, with a 6-h inter-trial interval. This version of the NOR, which depends on the functional integrity of the hippocampus [16], has previously been used to detect sex differences in the effects of enriched environment [12,57]. During Trial 1 (T1), the two objects in the arena were identical, and the time spent exploring them was recorded. During Trial 2 (T2), one of the objects was replaced by a new object (object N) that differed from the old one (object O) in terms of color, shape and texture, and, again, the time spent exploring each of the objects was recorded. The two identical objects used were metal, round-shaped, and white with colored stripes. The “novel” object was made of glass and was black and bottle shaped. A preference index was computed from *time spent exploring novel object (N)/total time spent exploring both objects (N + O)* in T2 and a discrimination index was computed by (N − O)/(N + O). Data acquisition and analysis was done using Kinoscope software [58,59]

#### 4.5.3. Tail Suspension Test

On the third day of behavioral testing, all mice were subjected to the Tail Suspension Test (TST), a screening tool for detecting the antidepressant potential of new compounds. All mice received TPC, SID or vehicle 30 min before the TST. Then, mice were hung from their tails and their behavior was video recorded for 6 min. Behavior was later scored by two experienced raters (NK, CD) using Kinoscope [58,59] and an automated computer algorithm (see below). The main behavioral outcome was immobility time, which is reduced by compounds that have antidepressant properties in humans [60].

#### 4.5.4. Automated Behavioral Analysis of TST

**Figure 9:**
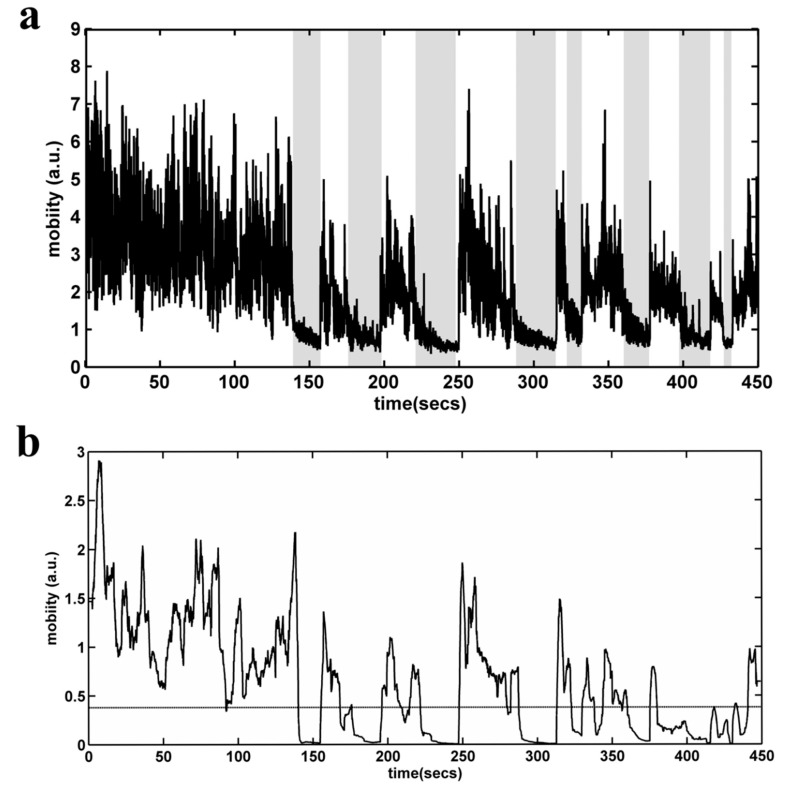
Automated behavioral analysis of the Tail Suspension Test. (**a**) Mobility time series (in arbitrary units) for each subject, constructed by calculating the absolute difference between each of the two subsequent gray level frames. Shaded regions define possible areas of false detection in the case of a simple threshold detector. (**b**) Mobility time series, after the application of a moving variance estimator in the form of a sliding window. The dashed line depicts the selected threshold.

TST analysis was performed by means of an automated video recognition algorithm. Initially, the user has to define a rectangular area of interest (AOI) in the video stream. Then, the AOI is converted to grayscale, and the absolute difference between each two subsequent gray level frames is calculated, in order to construct a mobility timeseries for each subject (Figure 9a). On each one of the produced timeseries, a moving variance estimator is applied in the form of a sliding window (Figure 9b). This approach has a two-fold purpose: first, we manage to enhance the information produced by the rapid movements of the mouse (project a maximum variance point, eliminating the response time—may be added by manual scoring) and, second, we avoid the false scoring that may occur when the rat swings to quietism (i.e., the shaded areas in Figure 9a). Finally, an adaptive threshold detector is applied in order to distinguish mobility and non-mobility conditions. The final outcome of the algorithm is the mobility time (in seconds) determined for a 6 min period. The immobility time is derived by subtracting total TST time (360 s) minus mobility time. The correlation of the algorithm scores with those of the two experienced raters (NK, CD) showed a very high degree of agreement.

#### 4.5.5. Analysis of Monoamines and Metabolites

At the end of the experiment, all mice were killed by rapid decapitation, their trunk blood was collected for serum chemistry analysis, their brain was excised, and the prefrontal cortex and the hippocampus was isolated, snap-frozen and stored at −80 °C until homogenization (see below). High-performance liquid chromatography (HPLC) detection of analytes was performed following a previously-described protocol [61,62], with minor modifications. In brief, the hippocampus and the prefrontal cortex were homogenized, deproteinized (in 500 μL of 0.1 N perchloric acid solution containing 7.9 mM Na_2_S_2_O_5_ and 1.3 mM Na_2_EDTA), and centrifuged (20,000 *g*, 45 min 4 °C), after which the recovered supernatants were stored at −80 °C until analysis. The analysis was carried out using a GBC LC1150 pump (GBC Inc, Braeside, VIC, Australia) and an Aquasil C18 column (150 mm × 2.1 mm; 5µm particle size; Thermo Fisher Scientific, Waltham, MA, USA) coupled to an electrochemical detector set at +800 mV (BAS LC4C, Bioanalytical Systems, West Lafayette, IN, USA). Reverse-phase ion pair chromatography (mobile phase: 50 mM phosphate buffer at pH 3.0, containing 300 mg/L sodium octylsulfate, 20 mg/L Na_2_EDTA, and acetonitrile added at a concentration of 6–9%) was used to assay noradrenaline (NA), dopamine (DA) and its metabolites 3,4 dihydroxyphenylacetate (DOPAC) and homovanillic acid (HVA), and 3-Methoxytyramine (3-ΜΤ), as well as serotonin (5-HT) and its metabolite 5-hydroxyindoleatic acid (5-HIAA). Quantification was done by comparing the areas under the curve (AUC) of external reference standards using the Clarity software (Data-Apex, Prague, Czech Republic). Additionally, an index of serotonergic and dopaminergic activity was obtained after calculating the turnover rates of 5-HT (5-HIAA/5-HT ratio) and DA (HVA/DA and DOPAC/DA ratios); these turnover rates reflect transmitter release and/or metabolic activity [40,63].

#### 4.5.6. Analysis of Aminoacids

The measurement of aminoacids in the prefrontal cortex and the hippocampus was performed using an LKB2248 pump (Pharmacia Biotechnology AB, Bromma, Sweden) coupled to a BAS LC4C electrochemical detector, after pre-column derivatization using a previously-described method with minor modifications [64,65]. Briefly, a glass–carbon working electrode (set at +800 mV), an Ag/AgCl_2_ reference electrode, and an Aquasil C18 (250 mm × 4.6 mm, 5 μm, from Thermo Fisher Scientific) were used. The mobile phase consisted of a 5% acetonitrile in 100 mM sodium-dihydrogen-phosphate buffer (pH 5.6), containing 0.65mM Na_2_EDTA. Samples were diluted to 5:1 with 0.1 M borax buffer (pH 10.4) to which o-phthalaldehyde was added; this mixture was left to stand at room temperature for 10 min prior to injection. Comparison of areas under the curve (samples vs. reference external standards) using the Clarity software allowed quantification of the aminoacids glutamate, serine, aspartate, glycine, glutamine, taurine, arginine and GABA, as previously described [56].

#### 4.5.7. Serum Chemistry Analysis

For serum extraction, the collected trunk blood was centrifuged at 2000 rpm for 10 min at 4 °C and stored at −80°C until analysis. Thereafter, serum samples from all mice were analyzed using commercial reagents (MLR, by Medicon Hellas, Athens, Greece) for the quantification of aspartate aminotransferase (AST/SGOT), alanine aminotransferase (ALT/SGPT), γ-glutamyltransferase (GGT), urea (UREA) and creatinine (CREAT). The enzymatic methods used and their respective detection limits were as follows: AST/SGOT, modified International Federation of Clinical Chemistry (IFCC), 3.7U/L; ALT/SGPT, modified IFCC, 2.8U/L; GGT, IFCC, 3.9U/L; Urea, Urease UV, 2.4 mg/dL; creatinine, Jaffe kinetic, 0.06 mg/dL.

#### 4.5.8. Statistical Analysis

The results were analyzed with SPSS v.24 (IBM Corp, NY, USA) after controlling so that all results met the data assumptions of ANOVA. The results for hippocampal NA, 5-HT, 5-HIAA, taurine, cortical HVA and DOPAC/DA ratio, SGPT and GGT were analyzed after cubic or cubic root transformation, to ensure homogeneity of variance. Group comparisons were performed using one-way ANOVA, with treatment as an independent variable (Vehicle, TPC Low, TPC High, SID Low, SID High) followed by Dunnet’s post-hoc pairwise comparison of treatment levels vs. vehicle. The regressors for the multivariate linear regression included treatment (SID, TPC or vehicle) and all dependent variables that showed statistically significant differences in the one-way ANOVA. Statistical significance was set at *p* < 0.05 and a trend was determined at *p* < 0.1. Results are reported as means ± standard deviation (S.D.) in Table A3 and graphed as means ± standard error of the mean (SEM). The number of animals per group are Vehicle N = 9, TPC Low Dose = 9, TPC High Dose N = 7, SID Low Dose N = 9, SID High Dose N = 7.

## 5. Conclusions

In conclusion, TPC seems to improve cognition and reduce anxiety and depressive behavior, possibly by decreasing glutamate and increasing serotonin turnover in the prefrontal cortex, respectively. SID exerts sedative effects in high doses in female mice. This promising evidence could clarify their purported “neutraceutical” properties in preventing and treating neurologic and psychiatric disorders, leading to a more informed development of dietary recommendations, including their appropriate use as dietary supplements. Both extracts act in the brain, but their indications, adverse effects, optimal dosage, as well as the exact mechanism of action related to these effects for each sex, remain to be clarified by preclinical and clinical studies.

Finally, there is an imperative need for new treatments for neuropsychiatric disorders, such as depression, anxiety and Alzheimer’s disease, which all present a wide range of affective and cognitive symptoms [66]. Therefore, in future studies, it would be worth investigating whether the specific compounds included in these extracts could have promising antidepressant, cognitive and/or anxiolytic properties.

## Figures and Tables

**Table 1:** Serum biochemistry. Serum biochemistry measurements for each treatment group. TPC Low Dose = 10 mg/kg, High Dose = 30 mg/kg, SID Low Dose = 50 mg/kg, High Dose = 150 mg/kg. An asterisk denotes a significant difference between a treatment group and the corresponding vehicle-treated control group.

	Vehicle	TPC Low Dose	TPC High Dose	SID Low Dose	SID High Dose
Serum Biochemistry	Mean ± S.D.	Mean ± S.D.	Mean ± S.D.	Mean ± S.D.	Mean ± S.D.
AST/SGOT	336.8 ± 157.4	157.4 ± 77.46 *	155.6 ± 60.22 *	208.0 ± 53.67 *	323.2 ± 101.3
ALT/SGPT	67.78 ± 19.12	65.11 ± 35.43	41.43 ± 13.76	79.44 ± 33.40	73.33 ± 26.12
γ-GT	9.89 ± 6.57	5.56 ± 2.40	7.29 ± 1.25	6.22 ± 4.44	7.33 ± 1.75
urea	60.78 ± 10.96	49.44 ± 3.91 *	45.86 ± 8.67 *	58.44 ± 8.63	61.00 ± 10.33
creatinine	0.71 ± 0.11	0.73 ± 0.09	0.73 ± 0.14	0.77 ± 0.10	0.70 ± 0.09

**Figure A1:**
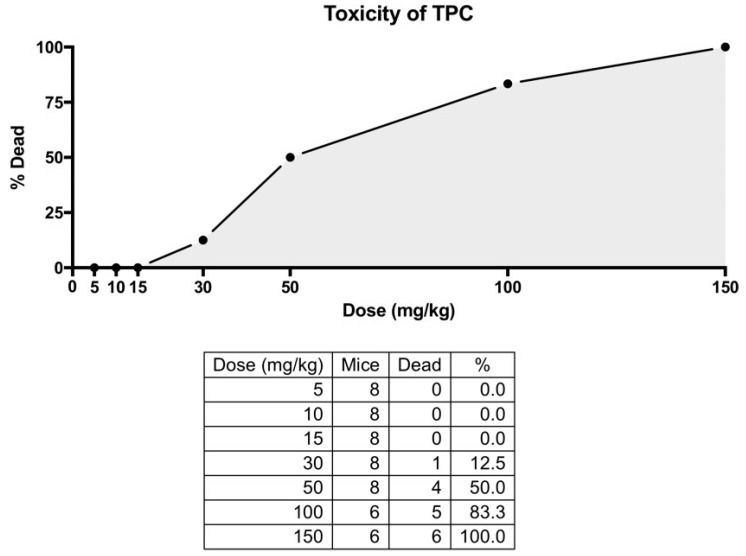
Analysis of Extract Toxicity.

## Data Availability

The authors confirm that the raw data supporting the findings of this study are available from the corresponding author upon request. The manuscript does not contain clinical studies or patient data.

## Appendix A

Table A1: Secondary metabolites identified in the TPC extract by UPLC-ESI(-)-HRMS/MS; Table A2: Secondary metabolites identified in the *S. scardica* water/ethanol extract by UPLC-ESI(-)- HRMS/MS; Table A3: Multivariate linear regression; Figure A1: Analysis of extract toxicity.

**Table A1:** Secondary metabolites identified in the TPC extract by UPLC-ESI(-)-HRMS/MS.

No.	Compounds	t_R_ (min)	Suggested Formula	[M-H]^−^ (m/z)	Error (ppm)	RDBeq	Ref.
1	Quinic acid	0.38	C_7_H_11_O_6_	191.0560	1.01	2.5	[67,68]
2	Citric acid	0.52	C_6_H_7_O_7_	191.0196	0.92	3.5	[68]
3	3,4-Dihydroxyphenylglycol	0.56	C_8_H_9_O_4_	169.0505	0.98	4.5	[67,68]
4	vanillic acid	1.22	C_8_H_7_O_4_	167.0349	1.07	5.5	[67]
5	**Hydroxytyrosol**	**1.56**	C_8_H_9_O_3_	153.0558	1.32	4.5	[67,68]
6	EDA	1.86	C_9_H_11_O_4_	183.0663	0.10	4.5	[67,68,69]
7	**EDA acid**	**1.92**	C_9_H_11_O_5_	199.0611	−0.28	4.5	[67,68,69]
8	Vanillin	2.23	C_8_H_7_O_3_	151.0402	1.22	5.5	[67,68]
9	Benzoic acid	2.34	C_7_H_5_O_2_	121.0298	2.70	5.5	[68]
10	p-Coumaric acid	2.59	C_9_H_7_O_3_	163.0402	1.19	6.5	[67]
11	Deoxy elenolic acid	2.90	C_11_H_13_O_5_	225.0765	−1.32	5.5	[67]
12	Hydroxylated form of elenolic acid (8R− or 8S−)	3.12	C_11_H_13_O_7_	257.0663	−1.54	5.5	[67]
13	Hydroxytyrosol Acetate	3.13	C_10_H_11_O_4_	195.0662	−0.47	5.5	[67]
14	Tyrosol	3.14	C_8_H_9_O_2_	137.0611	1.95	4.5	[67]
15	Hydroxylated form of elenolic acid (8R− or 8S−)	3.29	C_11_H_13_O_7_	257.0663	−1.54	5.5	[67]
16	**Elenolic acid (8R− or 8S−)**	**3.33**	C_11_H_13_O_6_	241.0715	−0.59	5.5	[67]
17	**Elenolic acid (8R− or 8S−)**	**3.24**	C_11_H_13_O_6_	241.0716	−0.58	5.5	[67]
18	Ferulic acid	3.49	C_10_H_9_O_4_	193.0505	0.98	6.5	[67]
19	Oleaceinic acid	3.58	C_17_H_19_O_7_	335.1129	−1.57	8.5	[3]
20	**Oleacein**	**3.79**	C_17_H_19_O_6_	319.1181	−1.82	8.5	[67,69]
21	Luteolin	3.95	C_15_H_9_O_6_	285.0400	−1.58	11.5	[67,68]
22	Elenolic acid methyl ester	4.08	C_12_H_15_O_6_	255.0871	−1.18	5.5	[67]
23	Oleocanthalic acid	4.15	C_17_H_19_O_6_	319.1184	−1.04	8.5	[67,69]
24	**Oleocanthal**	**4.43**	C_17_H_19_O_5_	303.1233	−1.47	8.5	[67,69]
25	Apigenin	4.49	C_15_H_9_O_5_	269.0454	−0.50	11.5	[67,68]
26	Diosmetin	4.67	C_16_H_11_O_6_	299.0555	0.50	11.5	[67,68]
27	**MFOA (8R− or 8S−)**	**4.93**	C_19_H_21_O_8_	377.1237	−1.17	9.5	[67]
28	**MFOA (8R− or 8S−)**	**5.02**	C_19_H_21_O_8_	377.1238	−1.01	9.5	[67]
29	MFLA (8R− or 8S−)	5.74	C_19_H_21_O_7_	361.1286	−1.87	9.5	[67]
30	Hydroxylinolenic acid	7.92	C_18_H_29_O_3_	293.2119	−1.05	4.5	[67]
31	MFLA (8R− or 8S−)	7.98	C_19_H_21_O_7_	361.1286	−1.79	9.5	[67]
32	Hydroxylinoleic acid	8.18	C_18_H_31_O_3_	295.2277	−0.71	3.5	[67]
33	Maslinic acid	8.20	C_30_H_47_O_4_	471.3477	−0.81	7.5	[67,68]
34	Hydroxyoleic acid	8.33	C_18_H_33_O_3_	297.2432	−0.97	2.5	[67,68]
35	Hydroxyoleic acid	8.47	C_18_H_33_O_3_	297.2431	−1.10	2.5	[67]
36	Octadecanedioic acid	8.51	C_18_H_33_O_4_	313.2380	−1.35	2.5	[67]
37	Hydroxylinoleic acid	8.60	C_18_H_31_O_3_	295.2276	−0.70	3.5	[67]
38	Oleanolic acid	8.97	C_30_H_47_O_3_	455.3530	−0.22	7.5	[67,68]
39	Betulinic acid	9.02	C_30_H_47_O_3_	455.3530	0.99	7.5	[67]

**Table A2:** Secondary metabolites identified in the *S. scardica* water/ethanol extract by UPLC-ESI(-)-HRMS/MS (with bold the major peaks of total ion chromatogram).

No.	Compounds	Rt (min)	Suggested Formula	[M-H]−m/z	Δm (ppm)	RDBeq	Ref.
1	Quinic acid	0.90	C_7_H_11_O_6_	191.0562	0.46	2.5	[70]
2	Malic acid	1.04	C_4_H_5_O_5_	133.0145	2.13	2.5	[70]
3	**Melittoside derivative**	**4.80**	C_22_H_33_O_17_	569.1723	0.07	15.5	[70]
4	**Chlorogenic acid**	**5.95**	C_16_H_17_O_9_	353.0870	−2.28	8.5	[70]
5	Neochlorogenic acid	6.03	C_16_H_17_O_9_	353.0868	−2.87	8.5	[71]
6	Apigenin 7-O-allosyl(1 → 2)-glucoside	6.39	C_27_H_29_O_15_	593.1512	0.19	13.5	[70,71]
7	Feruloylquinic acid	6.79	C_17_H_19_O_9_	367.1037	0.41	8.5	[70,71]
8	Echinacoside	7.04	C_35_H_45_O_20_	785.2510	−0.14	13.5	[70,71]
9	**Lavandulifolioside**	**7.08**	C_34_H_43_O_19_	755.2393	1.29	13.5	[71]
10	**Verbascoside**	**7.22**	C_29_H_35_O_15_	623.1981	0.75	12.5	[70,71]
11	Forsythoside B	7.31	C_34_H_43_O_19_	755.2382	−2.69	13.5	[70,71]
12	Samioside	7.36	C_34_H_43_O_19_	755.2379	−3.38	13.5	[70,71]
13	**Isoscutellarein 7-O-allosyl(1** **→** **2)-glucoside**	**7.36**	C_27_H_29_O_16_	609.1450	1.47	13.5	[70,71]
14	Luteolin 7-O-allosyl-(1 → 2)-[6″-O-acetyl]-glucoside	7.43	C_29_H_31_O_17_	651.1550	−2.61	14.5	[70,71]
15	Isoverbascoside	7.47	C_29_H_35_O_15_	623.1965	−2.56	12.5	[71]
16	Hypolaetin 7-O-[6‴-O-acetyl]-allosyl(1 → 2)-glucoside	7.54	C_29_H_31_O_18_	667.1516	0.68	14.5	[70,71]
17	3′-O-Methylhypolaetin 7-O-allosyl(1 → 2)-glucoside	7.57	C_28_H_31_O_17_	639.1567	0.77	13.5	[70,71]
18	Allysonoside (Isomer I)	7.58	C_35_H_45_O_19_	769.2561	0.30	13.5	[70,71]
19	Allysonoside (Isomer II)	7.71	C_35_H_45_O_19_	769.2532	−3.67	13.5	[70]
20	Leucosceptoside A	7.75	C_30_H_37_O_15_	637.2138	0.40	12.5	[70,71]
21	Apigenin 7-O-glucoside	7.78	C_21_H_19_O_10_	431.0984	0.82	12.5	[70,71]
22	Isoscutellarein 7-O-[6‴-O-acetyl]-allosyl(1 → 2)-glucoside	7.82	C_29_H_31_O_17_	651.1549	−3.09	14.5	[70,71]
23	Apigenin 7-O-[6‴-O-acetyl]-allosyl(1 → 2)-glucoside	7.91	C_29_H_31_O_16_	635.1598	−3.11	14.5	[70,71]
24	**Isoscutellarein 7-O-allosyl-(1 → 2)-[6″-O-acetyl]-glucoside**	**7.97**	C_29_H_31_O_17_	651.1551	−2.31	14.5	[70,71]
25	Chryseriol 7-O-[6‴-O-acetyl]-allosyl(1 → 2)glucoside	8.08	C_30_H_33_O_17_	665.1705	−2.77	14.5	[70,71]
26	**3’-O-Methylhypolaetin 7-O-[6‴-O-acetyl] -allosyl(1 → 2)-glucoside**	**8.15**	C_30_H_33_O_18_	681.1659	−1.98	14.5	[70,71]
27	4’-O-Methylhypolaetin 7-O-[6‴-O-acetyl] -allosyl(1 → 2)-glucoside	8.58	C_30_H_33_O_18_	681.1652	−2.96	14.5	[70,71]
28	Hypolaetin 7-O-[6‴-O-acetyl] -allosyl-(1 → 2) [6″-O-acetyl]-glucoside	8.64	C_31_H_33_O_19_	709.1602	−3.01	15.5	[70,71]
29	3′-O-Methylhypolaetin 7-O-[6‴-O-acetyl]-allosyl-(1 → 2)-[6″-O-acetyl]-glucoside or	8.82	C_32_H_35_O_19_	723.1756	−3.05	15.5	[70,71]
30	Luteolin 7-O-[6‴-O-acetyl]-allosyl-(1 → 2)-[6″-O-acetyl]-glucoside	8.84	C_31_H_33_O_18_	693.1652	−2.91	15.5	[70,71]
31	**Isoscutellarein 7-O-[6‴-O-acetyl]-allosyl(1 → 2)-[6″-O-acetyl]-glucoside**	**9.10**	C_31_H_33_O_18_	693.1661	−1.67	15.5	[70,71]
32	4′-O-Methylisoscutellarein 7-O-[6‴-O-acetyl]-allosyl(1 → 2)-glucoside	9.16	C_30_H_33_O_17_	665.1704	−2.87	14.5	[70,71]
33	**4′-O-Methylhypolaetin 7-O-[6‴-O-acetyl] -allosyl-(1 → 2) [6″-O-acetyl]-glucoside**	**9.26**	C_32_H_35_O_19_	723.1765	−1.87	15.5	[70,71]
34	**Apigenin 7-(6″-p-coumaroylglucoside)**	**9.73**	C_30_H_25_O_12_	577.1339	−2.15	18.5	[70,71]
35	Apigenin	9.79	C_15_H_9_O_5_	269.0451	−1.66	11.5	[70,71]
36	**Apigenin 7-(4″-p-coumaroylglucoside)**	**9.80**	C_30_H_25_O_12_	577.1339	−2.25	18.5	[70,71]
37	4′-O-Methylisoscutellarein 7-O-[6‴-O-acetyl]-allosyl-(1 → 2)-[6″-O-acetyl]-glucoside	10.28	C_32_H_35_O_18_	707.1808	−2.97	15.5	[70,71]
38	Luteolin derivative	11.06	C_17_H_13_O_6_	313.0710	−2.40	11.5	[70]
39	Hypolaetin trimethylether (Isomer I)	11.55	C_18_H_15_O_7_	343.0826	0.71	11.5	[70]
40	Hypolaetin trimethylether (Isomer II)	11.68	C_18_H_15_O_7_	343.0828	1.29	11.5	[70]

**Table A3:** Multivariate Linear Regression.

			Hipocampus				Prefrontal Cortex				
Behavior	Variance Explained	Glutamate	Aspartate	3-MT	5-HT Turnover	Glutamate	Aspartate	3-MT	5-HT Turnover	5-HIAA	Taurine
OF Horizontal Activity	43%	n.s.	n.s.	n.s.	n.s.	n.s.	n.s.	n.s.	0.002 *	n.s.	n.s.
Of Vertical Activity	43%	n.s.	n.s.	n.s.	n.s.	n.s.	n.s.	n.s.	0.011 *	n.s.	n.s.
OF Stereotypical Activity	46%	n.s.	n.s.	n.s.	n.s.	0.013 *	n.s.	n.s.	0.058	n.s.	n.s.
OF Entries in Center	35%	n.s.	n.s.	n.s.	n.s.	0.075	n.s.	n.s.	0.064	n.s.	n.s.
TST Immobility	19%	n.s.	n.s.	n.s.	n.s.	n.s.	n.s.	n.s.	n.s.	n.s.	n.s.
NOR Exploration Training	30%	n.s.	n.s.	n.s.	n.s.	n.s.	n.s.	n.s.	n.s.	n.s.	n.s.
NOR Exploration Testing	43%	n.s.	n.s.	n.s.	0.072	0.015 *	n.s.	n.s.	n.s.	0.076	0.022 *
NOR Preference Index	50%	0.050 *	n.s.	n.s.	n.s.	0.003 *	0.097	n.s.	n.s.	0.065	n.s.
NOR Discrimination Index	55%	0.048 *	n.s.	n.s.	n.s.	0.003 *	n.s.	n.s.	n.s.	0.082	n.s.

Following an initial observation during a pilot experiment, in which animals that received the TPC extract died, a toxicological study was performed to ascertain the appropriate doses of TPC and SID extracts. Animals received escalating single doses of TPC and SID intraperitoneally. For TPC, the administered doses were 5, 10, 15, 30, 50, 100 and 150 mg/kg. Due to the pre-observed toxicity of TPC at higher doses, fewer animals received the 100 and 150 mg/kg doses (n = 6 instead of n = 8). For SID, the doses administered were 5, 10, 100 and 1000 mg/kg (n = 8 for each dose). SID extract did not show any signs of toxicity or animal signs of distress in doses up to 1000 mg/kg. On the contrary, olive oil TPC induced significant toxicity at the stage of initial pilot administration. A more detailed toxicological study (Figure A1) determined that the LD80 for the intraperitoneal injection of TPC was 100 mg/kg and the LD50 was 50 mg/kg, with most deaths occurring within 48 h after TPC administration. Only one animal died at the dose of 30 mg/kg, one week after the administration of TPC, with no signs of suffering in between, so it is not clear whether this death was accidental or if it could be attributed to TPC. While natural and herbal compounds marketed as “neutraceuticals” are potentially harmful in both humans and animals, their underlying toxic mechanisms are largely unknown. Therefore, a toxicological screening is always necessary, before the investigation of their pharmacology [72]. Toxicity often manifests as liver or kidney injury. However, our serum biochemical measurements indicated that the low doses used in this study do not cause liver or kidney toxicity, and even exert a rather beneficial effect in serum biochemical parameters. Nevertheless, because of TPC toxicity during pilot administration, a toxicological study was performed (Figure A1). TPC lethal doses were LD50 = 50 mg/kg and LD80 = 100 mg/kg. To our knowledge, this is the first published in vivo toxicological study of olive oil or its extracts. Another study in *Drosophila* underlines that virgin olive oil did not cause mutations or recombinations, although lampante olive oil, a non-edible, low-quality byproduct, gave inconclusive results at high concentrations [73]. It can thus be hypothesized that TPC exerts a direct toxic effect, not mediated by gene mutations. However, TPC contains a multitude of compounds, making it difficult and rather impossible to pinpoint the single phenolic compound responsible and determine its exact mechanism of action. Regarding *Sideritis*, although previously found to be cytotoxic in cancer cell lines [74], our experiments did not reveal toxic effects (distress or death) in animals who received either the low or the high dose of the extract. This comes in line with two previous toxicological studies that proved the safety of *S. scardica* and *S. Taurica* [75,76].
